# Time-dependent extracellular matrix alterations of young tendons in response to stress relaxation: a model for the Ponseti method

**DOI:** 10.1098/rsif.2022.0712

**Published:** 2023-05-17

**Authors:** Mu-Huan Lee, Hung-Pei Tsai, Chris Lavy, Pierre-Alexis Mouthuy, Jan Czernuszka

**Affiliations:** ^1^ Department of Materials, University of Oxford, Parks Road, Oxford OX1 3PH, UK; ^2^ Division of Neurosurgery, Department of Surgery, Kaohsiung Medical University, Kaohsiung 80708, Taiwan; ^3^ Nuffield Department of Orthopaedics, Rheumatology and Musculoskeletal Science, Botnar Research Centre, University of Oxford, Windmill Road, Oxford OX3 7LD, UK

**Keywords:** clubfoot, Ponseti method, tendon, elastin, collagen crimp, stress relaxation

## Abstract

The Ponseti method corrects a clubfoot by manipulation and casting which causes stress relaxation on the tendons. Here, we examined the effect of long-term stress relaxation on tendon extracellular matrix (ECM) by (1) an *ex vivo* stress relaxation test, (2) an *in vitro* tenocyte culture with stress relaxation and (3) an *in vivo* rabbit study. Time-dependent tendon lengthening and ECM alterations including crimp angle reduction and cleaved elastin were observed, which illustrated the mechanism of tissue lengthening behind the treatment—a material-based crimp angle reduction resulted from elastin cleavage. Additionally, *in vitro* and *in vivo* results observed restoration of these ECM alterations along with increased elastin level after 7 days of treatment, and the existence of neovascularization and inflammation, indicating the recovery and adaptation from the tendon in reaction to the treatment. Overall, this study provides the scientific background and information that helps explain the Ponseti method.

## 1. Introduction

Congenital clubfoot is a complex paediatric foot deformity with an incidence of 1 in 1000 live births in the UK [1]. Left untreated, clubfoot leads to long-term disability, deformity and pain [1]. The Ponseti method is an effective treatment developed by Ignacio Ponseti in the 1940s [2]. This technique gradually corrects the deformity through a series of manipulations and casting in which the soft tissues around the foot are stretched. The strain is maintained by a plaster cast for approximately a week to allow adaptation and thus correction. This process is typically repeated about 6 times to fully correct the deformity [2].

In each cast, the tendons around the clubfoot would be the primary soft tissues to mechanically resist the correction acted upon [3,4]. The inflicted strain is estimated to be around 8% as demonstrated in electronic supplementary material, note S1. By contrast to the physiological loading regime (less than 4% strain) [5], this strain value is located at the beginning of the linear region of the mechanical profile (electronic supplementary material, figure S1). When a tendon is stretched and the strain is fixed to a value, stress relaxation will occur as a result [6]. At the same time, tissue remodelling could also occur to recover the structural, mechanical and functional properties of the tendon. Therefore, the stress relaxation lasting for days is controlled by not only the viscoelasticity [7] but also the biological adaptation of the tendon. In a tendon, tenocytes are the primary cells responsible for the extracellular matrix (ECM) turnover and remodelling [8–10] responding to the Ponseti method.

Studies on tendon stress relaxation have focused on the roles of collagen units [11,12], proteoglycans, and glycosaminoglycans [13]. However, the role of another important ECM component, elastin, in tendon stress relaxation has been unclear. Elastin accounts for only 1–2% of the tendon dry weight [14,15] and is believed to be responsible for inter-fibre recoil, transverse shear resistance, collagen crimp morphology and tissue retractability [16–18]. How elastin contributes to stress relaxation and, conversely, how stress relaxation alters elastin in a tendon are critical questions for tendon biomechanics and the improvement for the Ponseti method.

Although the Ponseti method is recognized as the standard treatment for clubfoot, the outcome of ECM integrity remains unknown. The current evaluation of the treatment outcome is based on clinical examinations and functional parameters such as Pirani score, Dimeglio scale, gait analysis, and kinematic variables [10,19]. Studies have attempted to image clubfoot with radiography and ultrasonography [20,21], but failed to reveal the microscopic details of the ECM. Micro-damage is likely to be produced as the tendon is forced to be strained for days.

In this work, we aim to study the effect of stress relaxation on tendon ECM to better understand the Ponseti method. An *ex vivo* stress relaxation was performed on bovine deep digital flexor tendons (DDFTs) under different durations, and the corresponding ECM alterations are reported. The cellular response of tenocytes to a long-term stress relaxation was examined *in vitro* using a dynamic culturing system. Lastly, an *in vivo* study modelling the Ponseti treatment was conducted on rabbit common calcaneal tendons (CCTs), i.e. the superficial digital flexor tendons (SDFTs) and gastrocnemius tendons (GTs).

## 2. Material and methods

### 2.1. Stress relaxation test

Four-week-old Dutch calf feet were obtained from a local abattoir. DDFTs of the feet were extracted at their origins and insertions, and stored at −80°C. Before testing, DDFTs were selectively air-dried on both ends, which left a 50–55 mm testing region in the centre, for secured gripping and failure prevention at the ends. The cross-sectional area was acquired from the width and thickness of the sample, assuming an oval shape for all samples. Three-dimensional-printed acrylonitrile butadiene styrene teeth were used to grip the dried sample ends firmly before inserting into a mechanical tester (Shimadzu Autograph AGS-X, 5 kN load cell). The initial stroke position was set to be at approximately 0.1 N of tensile force for each test. No preconditioning was performed before each test as it has not been reported nor has its purpose been validated in the Ponseti method [2]. Tendon samples were stretched under a strain rate of 0.5% s^−1^ until 8% engineering strain and then held for 0 h (immediate return to initial length), 2 h, 24 h and 48 h. During stress relaxation testing, the samples were wrapped with plastic films and sprayed with phosphate buffered saline (PBS) (Sigma, P4417) solution with 0.2 mg ml^−1^ sodium azide (Sigma, S2002) for every 4–6 h. After the test, tendons were removed from the tester and the tendon length was re-measured and compared with its original length. The lengthening percentage of the tendon tissue was calculated using the following equation:

2.1 $$\mathrm{Lengthening}\;(\text{\%})=\frac{\left(L-L_{0}\right)}{L_{0}}\times 100,$$

where *L*_0_ is the initial tendon length and *L* is the length after stress relaxation treatment. Control samples were prepared from the original tendon of each experimental group (0 h, 2 h, 24 h and 48 h) before the stress relaxation test.

### 2.2. Scanning electron microscopy

To prepare the samples for imaging, tendon samples were incubated in cryoprotectant solutions of ascending concentrations: 15% (w/v) sucrose (Sigma, S0389) and 15% (v/v) ethylene glycol (Sigma, 324558) in 0.1 M phosphate buffer (PB) (Sigma, S8282 and S7907) and 30% (w/v) sucrose and 30% (v/v) ethylene glycol in 0.1 M PB. Samples were then embedded in an optimal cutting temperature compound (OCT) (Labtech, 16-004004) before being transferred to a sliding microtome (Leica Biosystems, SM2000 R) for cryosectioning. Sectioned samples were fixed in 2.5% glutaraldehyde (Fluorochem, 358208), dehydrated by a series of ethanol solutions (50%, 70%, 80%, 90% and 100% × 3 times) and hexamethyldisilazane (Sigma, 440191) and coated with approximately 6 nm of platinum before imaging with an Evo MA10 SEM (Zeiss).

### 2.3. Immunohistochemistry

To prepare samples for fluorescent immunohistochemistry (IHC), formalin-fixed (Sigma, HT501128) tendon sections were permeabilized with 0.1% Triton X-100 (Sigma, T8787), blocked with 1% bovine serum albumin (BSA) (Sigma, A9647) and immunostained with rabbit anti-elastin antibody (Abcam, ab21607) as the primary antibody (1 : 200 dilution) and with Alexa Fluor™ 488 goat anti-rabbit IgG (Invitrogen, A-11034) as the secondary antibody (1 : 500 dilution). Cell nuclei were stained by incubating the samples in a DAPI solution (1 : 1000 dilution in PBS). Once staining was complete, samples were mounted on microscope slides with FluorSave mounting media (Sigma, 345789). The elastin morphology and cell nuclei were captured using an FV1000 confocal microscope (Olympus).

### 2.4. *In vitro* tenocyte culture with stress relaxation

To mimic the ECM environment of a tendon, decellularized tendon sections were used as scaffolds for cell culture. Briefly, bovine DDFTs of 3 cm in length were cryoprotected, embedded in OCT and cryosectioned into 300 µm thick sections. Decellularization was performed by incubating the sections in a 0.1% sodium dodecylsulfate (SDS) solution (Sigma, 74255) for 48 h and in a 200 U ml^−1^ deoxyribonuclease (DNase) (Sigma, D4527) solution at 37°C for 12 h. The SDS solution was in pH = 8 Tris buffer solution (Sigma, 252859), and the DNase solution was prepared in 1 M sodium chloride solution. The outcome of the decellularization is detailed in electronic supplementary material, note S2.

The customized setup for the tenocyte cell culture consists of a miniature linear motor, 304 stainless-steel grips, a stainless-steel chamber, a cell culture dish and a tendon scaffold seeded with tenocytes (electronic supplementary material, figure S2). Under this setup, a tension can be applied to the scaffold during a cell culturing experiment. Adult human tenocytes (OMB 0924) were extracted from an anterior cruciate ligament repair procedure in Nuffield Orthopaedic Centre with a Research Ethics Committee approval code: 09/H0606/11. Hamstring tenocyte cells were cultured from tissue explants and expanded using a culture medium containing Dulbecco's modified Eagle medium F12 (Lonza/SLS, LZBE12-719F), 10% fetal bovine serum (Labtech International, FCS-SA/500-41213) and 1% penicillin–streptomycin (Life Technologies, 15070063). The scaffolds (*n* = 3) were sterilized in a culture hood using a series of ethanol solutions (25%, 50% and 70%). Once the scaffolds were fixed on the grips in a warm sterile PBS bath, the scaffolds were sterilized again in 70% ethanol. The same setup was adopted in the control group with no tension being applied during the whole experiment. After washing with sterile PBS, the scaffolds were immersed in the culture medium. 100 000 cells (passage 2) were seeded dropwise onto each scaffold with a seeding volume of 63.5 µl. Once the cells had attached to the surface of the tendon scaffolds, additional culture medium (25 ml) was added to each group to allow full immersion. Cells were allowed to stabilize in the incubator for 20 h before a strain of 8% was applied using Zaber Console software. Culture medium was exchanged every 48-h interval. The whole culturing experiment lasted 21 days. Tendon samples were imaged with scanning electron microscopy (SEM) using an Evo MA10 SEM (Zeiss).

### 2.5. RNA extraction, reverse transcription and quantitative polymerase chain reaction

RNA was extracted and isolated using a Direct-zol RNA MicroPrep Kit (Zymo Research R2061, USA) following the provided protocol. The concentrations of the purified RNA samples were measured using a Nanodrop UV–visible spectrophotometer and only samples with 260/280 value greater than 1.6 were included. A High Capacity cDNA Reverse Transcription kit (Applied Biosystems, 4368814) was used to perform a reverse transcription. Following the provided protocol, a final volume of 20 µl of sample solution was used to create cDNAs.

The quantitative polymerase chain reaction (qPCR) was performed using a ViiA7 real-time PCR machine (Applied Biosystems, USA). Final cDNA was diluted to 1.25 ng µl^−1^ and 4 µl was used in a 10 µl reaction with Fast SYBR Master Mix (Applied Biosystems, 43856) according to the manufacturer's protocol. Glyceraldehyde 3-phosphate dehydrogenase (GAPDH) (Primerdesign) was used as an endogenous reference gene. QuantiTect primers for COL1A1 (Primerdesign) and ELN (Qiagen) were used in the qPCR. Gene expression was calculated using the comparative CT method according to Schmittgen & Livak [22].

### 2.6. *In vivo* rabbit model

The animal work was conducted at Master Laboratory Co. Ltd, Animal Laboratory, Taiwan. Experimental procedures using animals were performed in accordance with the Institutional Animal Care and Use Committee (IACUC) guidelines (IACUC number: 20T10-10).

**Figure 1.**
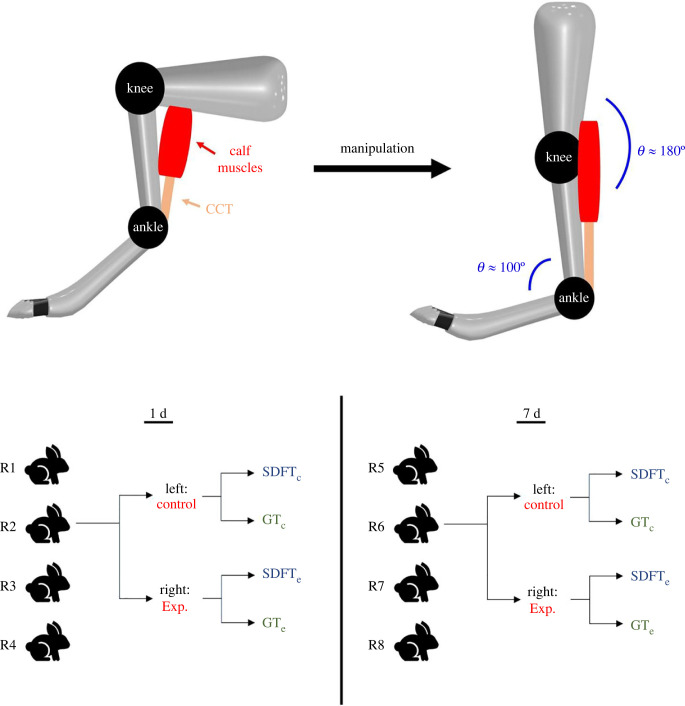
Illustrative images of the experimental design of the rabbit study. (*a*) Demonstration for the manipulation procedure to produce an engineering tensile strain of 8% on the rabbit CCT. (*b*) Experimental layout of the rabbit study displaying the experimental groups (1 d and 7 d), tendons examined (GT and SDFT), and the control sample selection.

To model the effect of a Ponseti cast on a tendon, rabbit CCTs were tested as numerous studies have applied them to study tendon repair and tendinopathy associated with mechanical treatment [23–25]. The correcting manipulation for hindfoot equinus, which is the last correction for clubfoot, was chosen for the model. Three adult New Zealand white (NZW) rabbits were used for the preliminary experiments and measurements. Like the situation in a human equinus correction where the Achilles tendon or the CCT is stretched, the CCT of a rabbit foot was also stretched by manipulation, and the resulting engineering strain of the CCT was measured. To perform the measurement, after the rabbit was anesthetized by the injection of Telazol (50 mg ml^−1^) into the muscles of the rabbit (0.12 ml kg^−1^), the fur of the foot was shaved with an electric animal shaver, and the skin covering the calf muscle and the CCT was removed using surgical blades. Two markings were made using a marker pen to create two reference points. The initial length (*L*_0_) between the two reference points was measured before stretching. Through manipulation, the knee was straightened while the foot was slowly dorsiflexed to an angle of approximately 100^o^ to create an elongation and tension on the CCT as shown in figure 1*a*. The final length (*L*) of the two reference points on the CCT due to this manipulation was measured. The mean strain ($\bar{\varepsilon }$) (*n* = 6) created by the manipulation was calculated using the equation below (equation (2.2)) to produce a strain value of approximately 8% on the CCT:

2.2 $$\bar{\varepsilon }=\frac{\sum_{i}^{n}((L_{i}-L_{0,i})/L_{0,i})}{n}.$$

Eight 45-day-old NZW rabbits were used in the animal study (figure 1*b*). Following the above-mentioned manipulation, every manipulated foot was fixed with taping. This procedure was performed without anaesthesia. The progression of the dorsiflexion of the rabbit foot would be stopped and fixed if the animal displayed any sign of pain during the process. For every rabbit, only one foot received manipulation and taping while the other foot was left untreated (control). Taping was maintained for 2 different durations: 1 day and 7 days before euthanasia with CO_2_. Four rabbits (*n* = 4) were studied in each duration group. Both the SDFT and GT of a CCT were dissected and analysed.

Tendon samples collected for SEM and IHC were prepared as described earlier. SEM imaging was conducted using a JSM-6360 SEM (JOEL). For IHC, sections were permeabilized with 0.1% Triton X-100 (Sigma, T8787) and blocked with 1% BSA (Merck, P02769) and 1% goat serum (Sigma, G9023). Elastin was immunostained with rabbit anti-elastin antibody (Abcam, ab21607) as the primary antibody and with goat anti-rabbit IgG (H + L)-FAM (LEADGENE, 21001) as the secondary antibody. Cell nuclei in the tendon were stained during mounting using a DAPI mountant (Sigma, F6057). Fluorescence imaging was performed using an LSM 700 confocal microscope (Zeiss).

### 2.7. Histology

Tendon tissues fixed in 10% formalin were transported to BioLASCO Taiwan Co. Ltd for sample preparation for histological staining. Briefly, tendon samples were dehydrated using a Histo-Tek VP1 vacuum tissue processor (Sakura), cleared with xylene (BURNETT, 00015B1) and embedded in paraffin. Tissue blocks were sectioned into 4 µm thick sections using a microtome (Amos Scientific, AEM460). H&E staining (Leica Biosystems, 3801522 and 3801602) was then performed on the tendon sections using a DRS-2000 automated slide stainer (Sakura). One section was stained and imaged for each tendon of each rabbit. Imaging was done by an optical microscope (Olympus).

### 2.8. Image analysis

Image analysis was performed on images derived from SEM, IHC and H&E staining using ImageJ to compare the ECM alterations between the stress-relaxed tendons and the controls.

Crimp analysis was conducted to compare the differences in crimp angle (*θ*) and crimp fibre side length (*d*) after stress relaxation (*ex vivo* bovine DDFTs and *in vivo* rabbit CCTs) as illustrated in electronic supplementary material, figure S3. For each experimental group, 30 measurements (*n* = 30) were recorded for both *θ* and *d*, and the values were compared to those obtained from the corresponding control. Two-sample *t* test or 1-way ANOVA and Tukey's *post hoc* test were conducted to test the hypothesis of no difference between the experimental groups.

For quantification of the cleaved elastin level, the two dimension cleaved elastin ratio (i.e. the area of cleaved elastin over the total area of the image) was calculated. Briefly, as illustrated in electronic supplementary material, figure S4, (1) the regions containing cleaved elastin were selected and cropped from the original image; (2) the cropped images were converted to 8-bit greyscale; (3) the threshold of the image was adjusted based on the mean fluorescence level of the local healthy/intact elastin (mean of 5 intensity maxima) where all fluorescence signals lower than this mean were excluded; and (4) the total area of the cleaved elastin was measured using particle analysis where only particles of size larger than 5 pixel^2^ were included. For each group, the mean cleaved elastin ratio of 20 images was calculated (*n* = 20).

To compare the elastin remodelling of CCTs between rabbits stress-relaxed for 1 day and 7 days, the level of elastin protein for each sample was also quantified by measuring the mean fluorescence intensity (*I*) of the images (*n* = 16) of each experiment. Following the equation

2.3 $$\frac{\Delta I_{7d}}{\Delta I_{1d}}=\frac{(I_{7d}-I_{7d,\mathrm{ctrl}})/I_{7d,\mathrm{ctrl}}}{(I_{1d}-I_{1d,\mathrm{ctrl}})/I_{1d,\mathrm{ctrl}}}\;,$$

for each type of tendon, the intensity difference between the treatment group and the control normalized by the control intensity was first calculated. The calculated intensity differential ratio of 7 days to 1 day was then derived.

Quantification of vascularity of the rabbit tendon ECM was performed on images acquired from H&E staining. The ratio (*R*_v_) of the area sum of vascular regions within the ECM over the total ECM area was derived for every single tendon section. The change of vascularity (Δ*V*) due to the stress relaxation treatment was then calculated using the following equation:

2.4 $$\Delta V=\frac{\left(R_{v,\mathrm{stress}-\mathrm{relaxed}}-R_{v,\mathrm{ctrl}}\right)}{R_{v,\mathrm{ctrl}}}\times 100\text{\%}.$$

Quantification of cell number in the rabbit tendon ECM was performed on images acquired from DAPI-stained sections. The mean number of cells (*n* = 10) captured over the total area of an image (*N*) was derived using particle analysis in ImageJ for each rabbit. The change of cell number (Δ*N*_cell_) due to the stress relaxation treatment was then calculated using the following equation:

2.5 $$\Delta N_{\mathrm{cell}}=\frac{\left(N_{\mathrm{stress}-\mathrm{relaxed}}-N_{\mathrm{ctrl}}\right)}{N_{\mathrm{ctrl}}}\times 100\text{\%}.$$

## 3. Results

### 3.1. Tendon exhibited lengthening, reduced stresses without loss of modulus and shortened toe region after a stress relaxation in 2 h

**Figure 2.**
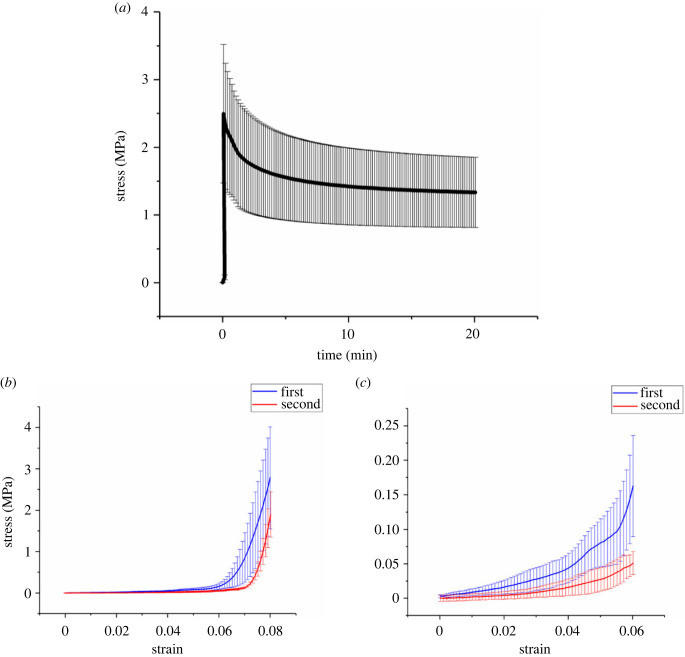
Mechanical profiles of the young bovine DDFTs which underwent 2 h of stress relaxation. (*a*) The stress–time curve of the initial 20 min of the tendon stress relaxation. (*b*) The stress–strain curves of the first (blue) and second (red) tensile straining. The second test was conducted at the same initial stroke position and under the same strain rate of 0.5% s^−1^ as the first test. (*c*) The stress–strain curve of the initial 6% strain of (*b*). All data are displayed as the mean ± standard deviation (s.d.) (*n* = 3).

The stress–time profile of the stress relaxation test of the 2 h experimental group is displayed in figure 2*a*. As an illustration, all experimental groups produced similar stress–time profiles in which the stress response clearly differentiated the toe and linear regions in the first 16 s under a slow strain rate of 0.5% s^−1^ and then exponentially decayed over time after *t* = 16 s where 8% strain was reached.

**Figure 3.**
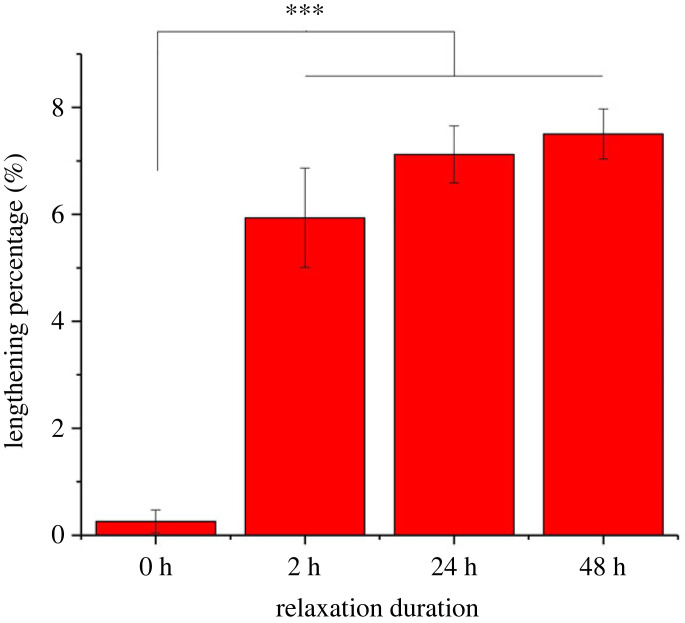
Tendon lengthening percentages due to stress relaxation. The percentages of tendon lengthening of different relaxation durations (0 h, 2 h, 24 h and 48 h) calculated based on equation (2.1). All data are displayed as the mean ± s.d. (*n* = 3). Significant differences were analysed by Tukey's *post hoc* test. *p < 0.05, ***p < 0.001.

After the stress relaxation treatment, the length of DDFT samples has permanently increased. Figure 3 shows the lengthening percentages of different relaxation durations in which the degree of lengthening is dependent on the duration of relaxation. No statistical difference was spotted between the 24 h and 48 h groups implying the maximum level of lengthening was achieved within the first day. Stress relaxation for 2 h was able to reach approximately 83% of this maximum lengthening value. While the actual time required to produce this lengthening value is still unclear, it may fall within the first few hours of the treatment which is much shorter than the duration of a single Ponseti cast.

To investigate the variation in mechanical profile, a second tensile test (starting at the same initial stroke position) was conducted under the same condition on the 2 h group immediately after the stress relaxation. The resulting mean stress–strain curve along with the initial curve are displayed in figure 2*b*,*c*. Throughout the whole 8% elongation, the stresses of the second tensile test were lower than those of the first test. This reduction of stresses likely resulted from the long-term stress relaxation, causing plastic deformation of certain ECM components in the tendon. However, the Young's modulus, which was derived by curve-fitting the linear region below 8% strain, did not decrease statistically (*p* < 0.05 from paired t test) (from 201.6 ± 62.3 to 250.7 ± 68.3 MPa), suggesting no significant damage was inflicted within the collagen fibres. Besides the deviations in stresses, the toe region was reduced, and the transition from toe to linear region became sharper after the stress relaxation as shown in figure 2*b*,*c*. For the second curve, the actual starting point is about 5–6% strain on the *X*-axis where detectable stress values appeared, meaning the toe region only comprised about 1–2% compared to approximately 6% in the first curve.

### 3.2. Long-term stress relaxation to model the Ponseti method causes time-dependent collagen crimp angle reduction

**Figure 4.**
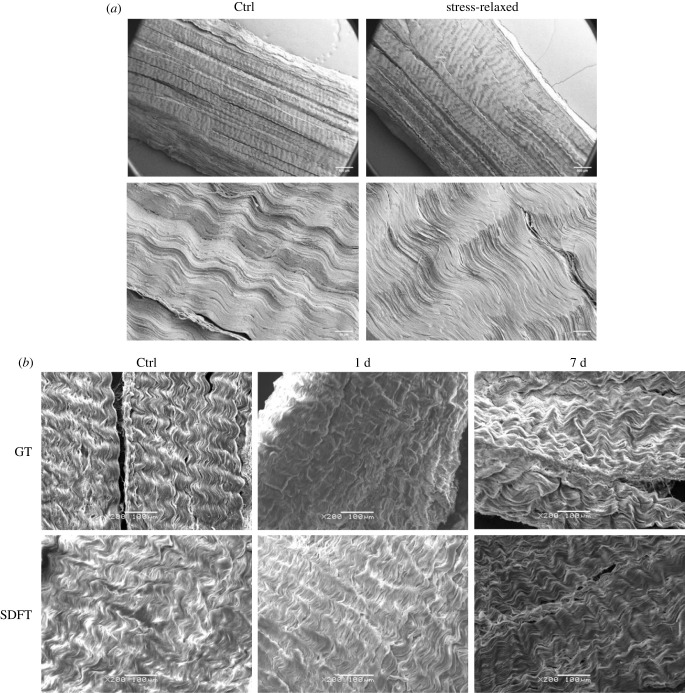
SEM images of the tendon sections. Tendon surface morphology of: (*a*) the young bovine DDFT sections with or without the treatment of stress relaxation; (*b*) the young rabbit GT (top row) and SDFT (bottom row) sections of the control (left column), the foot stress-relaxed for 1 d (middle column), and the foot stress-relaxed for 7 d (right column).

**Figure 5.**
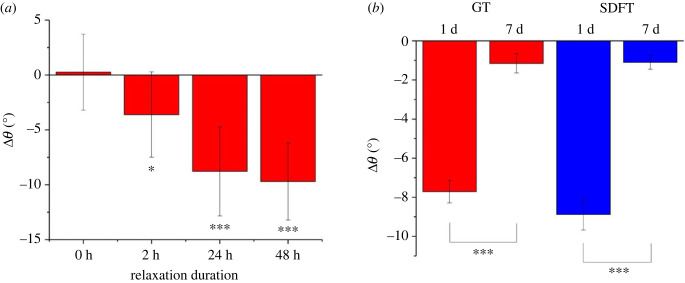
Summary of the values of crimp angle reduction due to stress relaxation. (*a*) The amount of the crimp angle reduced in a young bovine DDFT after the treatment of stress relaxation plotted over different relaxation durations (0 h, 2 h, 24 h and 48 h). Data are displayed as the mean ± s.d. (*n* = 30). (*b*) The amount of the crimp angle reduced after the treatment of stress relaxation plotted over different relaxation durations (1 d and 7 d) for both GT and SDFT of the young rabbits. Data are displayed as the mean ± s.d. (*n* = 4). Each Δ*θ* value is calculated by subtracting the stress-relaxed angle from the mean angle of the control (*θ*_ctrl_ − *θ*_exp_). Significant differences were analysed by 2-sample *t*-test. *p < 0.05, ***p < 0.001.

The collagen structure of bovine DDFTs treated with stress relaxation is shown in figure 4*a*. Collagen crimp was visibly flattened in the stress-relaxed sample compared to the control. As no tension was applied during sample preparation and imaging, this crimp angle reduction was irreversible. Quantification of the crimp angle reduction (Δ*θ*) of DDFT is shown in figure 5*a*. No statistical difference was seen on fibre side length (electronic supplementary material, figure S5). From figure 5*a*, different durations of stress relaxation resulted in different levels of crimp angle reduction which coincides with the levels of the tissue's lengthening (2 h < 24 h = 48 h). No angle reduction was spotted statistically in the 0 h group which highlighted the importance of relaxation duration.

SEM results from the rabbit study, as shown in figure 4*b*, also discovered crimp angle reduction. Both the GTs and SDFTs stress-relaxed by manipulation and taping for 7 days appeared to show lower levels of crimp angle reduction compared to rabbits stress-relaxed for 1 day. The mean Δ*θ* values are shown in figure 5*b*. For both the GT and SDFT, lower Δ*θ* values were seen in the 7 d group compared to the 1 d group. Unlike the *ex vivo* stress relaxation experiment on bovine DDFTs, the Δ*θ* value for the 1 d experimental group was slightly lower for the rabbit tendons. Furthermore, instead of plateauing after 1 day, Δ*θ* values decreased over time (day 7), suggesting the possibility of the restoration of collagen crimp.

### 3.3. Alterations of elastin in tendon extracellular matrix and tenocyte gene expressions due to stress relaxation

**Figure 6.**
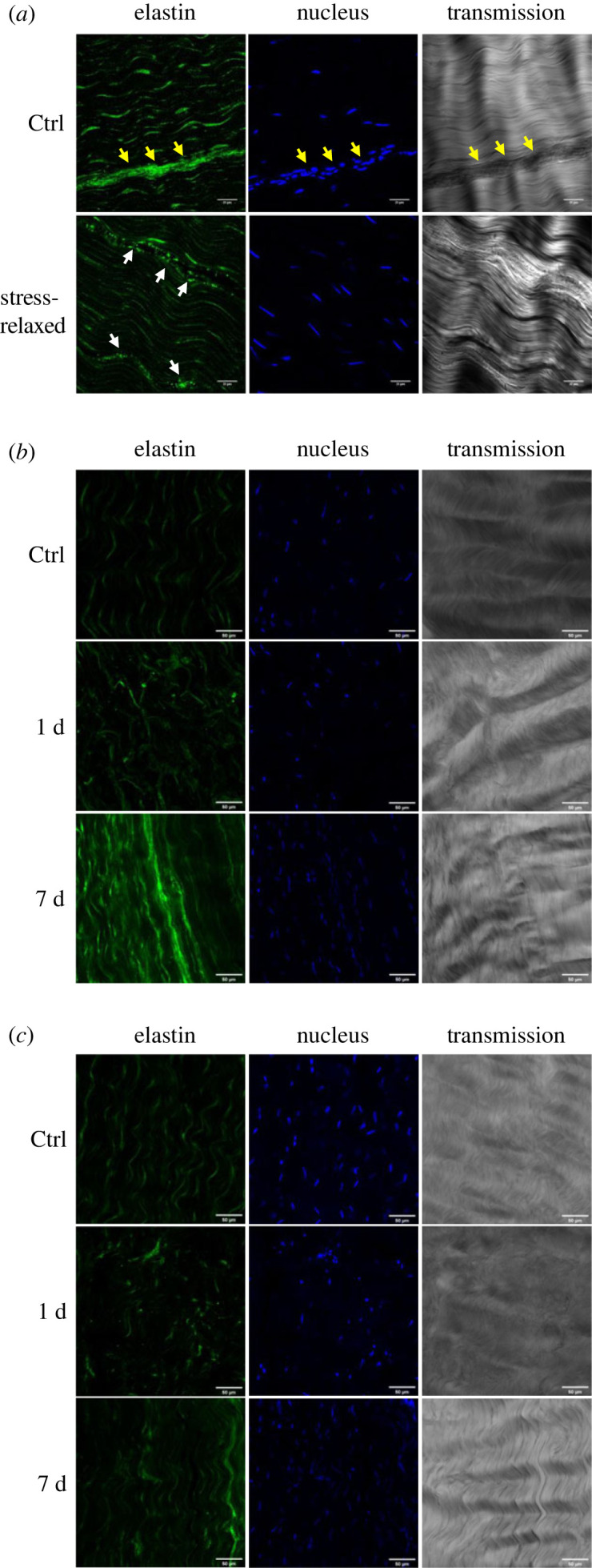
Fluorescence images of immunostained elastin and DAPI-stained cell nuclei. Representative immunostained tendon images captured by confocal microscopy revealing the morphology of elastin (green), cell nuclei (blue) and tendon matrix (grey) in (*a*) a bovine DDFT, (*b*) a rabbit GT, and (*c*) a rabbit SDFT. IFM is marked with yellow arrows. Examples of cleaved elastin caused by stress relaxation (stress-relaxed, 1 d and 7 d) can be seen in (*a*) (marked with white arrows), (*b*) and (*c*) appearing as aggregated clusters with higher fluorescence intensity. No elastin cleavage can be found in the controls of (*a*), (*b*) and (*c*).

**Figure 7.**
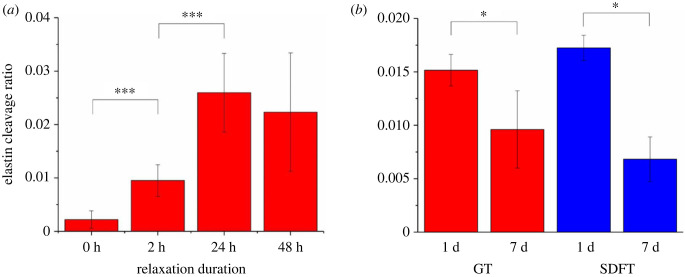
The elastin cleavage ratio in the tendon matrix after the treatment of stress relaxation. (*a*) Values of cleaved elastin ratio of young bovine DDFTs treated with stress relaxation of different durations (0 h, 2 h, 24 h and 48 h). Data are displayed as the mean ± s.d. (*n* = 20). (*b*) Values of cleaved elastin ratio of young rabbit GTs and SDFTs treated with stress relaxation for 1 d and 7 d. The elastin cleavage ratio is defined as the total area of cleaved elastin over the image area (tendon matrix area) for each fluorescence image. Data are displayed as the mean ± s.d. (*n* = 4). Significant differences were analysed by Tukey's *post hoc* test or 2-sample *t*-test. *p < 0.05, ***p < 0.001.

IHC revealed the morphology of elastin in the tendon ECM (figure 6). For both the controls of bovine DDFTs (figure 6*a*) and rabbit CCTs (figure 6*b*,*c*), within a tendon fascicle matrix (FM), elastin conformed to the collagen crimp pattern and was often localized around tenocytes. In the regions of IFM (marked with yellow arrows in figure 6), the elastin does not appear as aligned/organized fibres but rather as clusters spreading all over the IFM. Additionally, the emission intensity of elastin in the IFM was much higher, demonstrating a higher amount/density of elastin in the IFM. Stress-relaxed bovine and rabbit tendons showed cleavage of elastin within the FM. The features of cleaved elastin include: (1) cluster-forming cleaved elastin (mostly spherical shape); (2) assembly or localization of these clusters; (3) higher elastin fluorescence intensity from these clusters compared to the surrounding healthy elastin. The level of elastin cleavage was quantified and is displayed in figure 7. A time-dependency (2 h < 24 h = 48 h) similar to tissue lengthening and crimp angle reduction was spotted in elastin cleavage ratio of bovine DDFTs (figure 7*a*).

Elastin cleavage was also observed in stress-relaxed rabbit tendons. From figure 6*b*,*c*, tendons treated for 7 d showed (1) less cleaved elastin, (2) larger regions of intact elastin fibres and (3) higher overall elastin fluorescence intensity compared to the 1 d group. Quantification of cleaved elastin (figure 7*b*) also found decreased values of cleavage ratio in the 7 d group for both GT and SDFT, which again coincides with the findings in crimp angle reduction, i.e. lower Δ*θ* corresponds to lower cleaved elastin ratio. The decrease in cleavage ratio implies potential healing/remodelling taking place after day 1. Nevertheless, compared to the results of crimp angle reduction, smaller gaps were seen between the cleavage ratios of the 1 d group and the 7 d group for both GT and SDFT.

**Figure 8.**
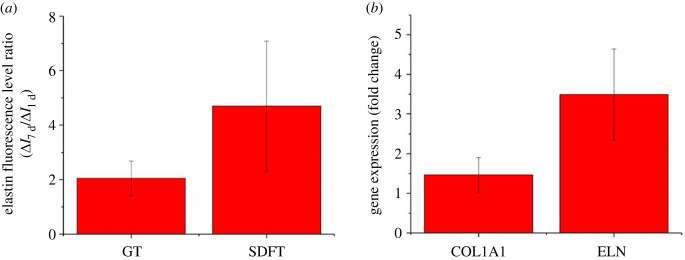
Elastin and collagen type I expression levels due to the treatment of stress relaxation. (*a*) Elastin protein level ratios of 7 d stress relaxation over 1 d stress relaxation of young rabbit GTs and SDFTs calculated based on equation (2.3) using fluorescence level changes normalized by the controls. Data are displayed as the mean ± s.d. (*n* = 4). (*b*) Fold changes of gene expressions of COL1A1 and ELN of human tenocytes cultured on decellularized tendon treated with 21 d stress relaxation compared to tenocytes without stress relaxation. Data are displayed as the mean ± s.d. (*n* = 3 for COL1A1 and *n* = 2 for ELN). One data point of ELN level was excluded as an outlier. The complete data are shown in electronic supplementary material, table S1. Significant differences were analysed by 2-sample *t*-test. No statistical difference was found in all groups in both experiments.

The change of elastin level in the ECM of rabbit CCTs was quantified based on the fluorescence intensity from IHC. As shown in figure 8*a*, for both tendon types, the increase of elastin fluorescence level of the 7 d group was 2–4 times higher than that of the 1 d group. Results from the qPCR (figure 8*b*) also show upregulation on both gene expressions of COL1A1 and ELN from human tenocytes stress-relaxed for 21 days compared to the control.

### 3.4. Changes in vascularity, increased cell number and incidence of inflammation were observed in the tendon extracellular matrix after stress relaxation *in vivo*

**Figure 9.**
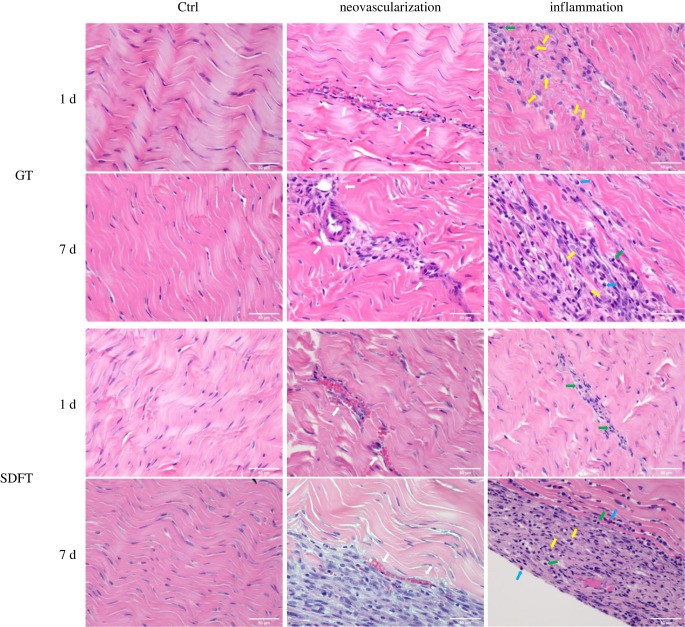
H&E-stained GT and SDFT. Optical images of rabbit GTs and SDFTs treated with 1 d and 7 d stress relaxation from H&E staining. A control image of each group is displayed in the 1st column. The collagen matrix displaying the usual wavy crimp pattern was stained pink, and the cell nuclei were stained purplish blue. Representative regions of neovascularization (2nd column) and inflammation (3rd column) of the tendon FM are displayed for each experimental group. Neovascularization (marked with white arrows) and inflammatory cells including macrophages (marked with yellow arrows), heterophils (marked with blue arrows), and lymphocytes (marked with green arrows) could be observed.

**Figure 10.**
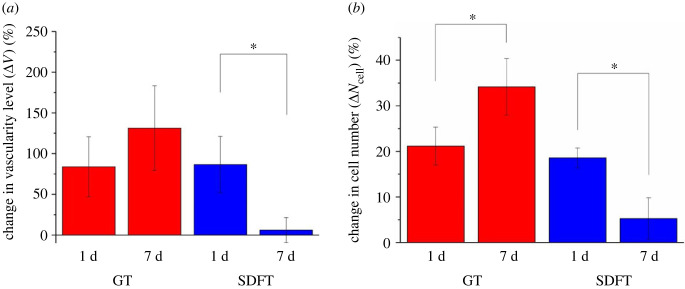
Quantification of vascularity and cell number alterations due to stress relaxation. (*a*) Changes in vascularity level of the rabbit GTs and SDFTs after 1 d and after 7 d compared to their corresponding controls were quantified based on equation (2.4). (*b*) Changes in cell numbers in the rabbit GTs and SDFTs after 1 d and after 7 d compared to their corresponding controls. Data are displayed as the mean ± s.d. (*n* = 4). Significant differences were analysed by 2-sample *t*-test. *p < 0.05.

Images from H&E staining are shown in figure 9. The control sections of all rabbit tendons display a healthy tendon ECM composed of pink wavy collagen matrix within which tenocytes reside. On the other hand, stress-relaxed GTs and SDFTs (figure 9) showed apparent ECM alterations including (1) increased vascularity or neovascularization and (2) migration/infiltration of inflammatory cells (e.g. macrophages, heterophils and lymphocytes which are marked with yellow arrows, blue arrows and green arrows, respectively). The existence of inflammation was seen in both day 1 and day 7 for both tendon types. Quantification of vascularity changes (Δ*V*) is shown in figure 10*a*. Both tendon types display similar increase in vascularity level (Δ*V* ≈ 80%) after 1 day of treatment compared to the untreated controls. While Δ*V* for the 7 d group was higher than that for the 1 d group in the GTs (+56.7%), no statistical difference was found between them. For the SDFTs, Δ*V* was statistically lower at day 7 (−92.9%). Regarding the change in cell number (Δ*N*_cell_) shown in figure 10*b*, GT displayed an increase in Δ*N*_cell_ (+61.4%) in day 7 compared to day 1; while SDFT displayed a decrease in Δ*N*_cell_ (−71.7%) in day 7 compared to day 1.

## 4. Discussion

A long-term stress relaxation modelling the Ponseti method successfully lengthened a tendon without the loss of elastic modulus *in vitro* in less than 24 h. From the *ex vivo* bovine results, 83% of the maximum lengthening value was achieved in 2 h of treatment. Generally, several hours of time is not sufficient for ECM remodelling to lengthen the tendon [26,27], suggesting the lengthening response from the tissue would also be a material-based adaption in an actual Ponseti treatment. It also shows that the casting duration may be shortened in the actual treatment [28,29]. However, several ECM alterations observed in this study may lead to functional and health complications for a tendon.

In both the *ex vivo* bovine and *in vivo* rabbit experiments, crimp angle reduction (figure 4) and elastin cleavage (a degeneration seen in aged elastic tissues [30–32]) (figure 6) are the two major ECM alterations observed, and they are likely to be the mechanism behind the Ponseti method. To our knowledge, this is the first work to report cleaved elastin in a tendon after mechanical treatment. When the elastin protein was clipped and cleaved, the hydrophobic domains in elastin exposed in the hydrophilic environment would result in folding and clustering of the protein to lower the free energy state [33–35]. These clusters of elastin can be seen in the strained tendons in both the *ex vivo* and *in vivo* experiments (figure 6). With no living cell present in the tendon in the *ex vivo* experiment, these cleaved elastin clusters were caused by the mechanical treatment rather than elastin remodelling. A time-dependency (2 h < 24 h = 48 h) was seen in tendon lengthening (figure 3), crimp angle reduction (figure 5) and cleaved elastin (figure 7), indicating an association between them. Since the elastin is known to be responsible for maintaining the crimp morphology, elastin cleavage would result in irreversible reduction of crimp angle under tension. A reduced crimp angle also produced a mechanical profile with reduced toe region which contributed to the permanent lengthening of the tendon.

Crimp, a unique planar sinusoidal waviness of the collagen fibres, plays a vital role in the mechanical behaviour of tendon including natural shock-absorbing, elastic recoil and physiological toe region [36]. Changes in crimp morphology such as crimp size, crimp angle and crimp periodicity length are known to be influenced by tendon types, age, training, injuries and even pathological conditions [37–39]. Generally, for a tendon, ageing, injuries, and training would result in the reduction of crimp angle [38,39]. Franchi *et al*. [39] stated that the crimp angle reduction is a consequence of a functional adaptation of the ECM to exercise to increase tendon stiffness and force transmission efficiency; however, in the case of an aged or injured tendon, a reduced crimp angle would predispose the fibres to overstretching and further damage [40,41]. The microtrauma could accumulate with further high load cycles and eventually result in tendonitis [40,41]. While health complications associated with cleaved elastin have been reported in cardiovascular system [42,43], no work has studied elastin cleavage in a tendon to our knowledge. Due to the poor turnover rate and remodelling capability of elastin in matured tissues, they are generally irreparable for adults when damaged [30], and the body tends to recover the affected region by deposition of collagens or proteoglycans [44,45], which would cause the elastic tissue to lose its extensibility and reversible recoil [46]. Hence, treating clubfoot at a younger age may be a potential criterion for the Ponseti method as the elastin turnover is much more dynamic before adolescence [47]. In addition, cleaved elastin peptides or elastin-derived peptides (EDPs) have been reported to regulate cellular activities [48,49]. However, EDPs are also believed to promote the progression of chronic inflammation [50].

**Figure 11.**
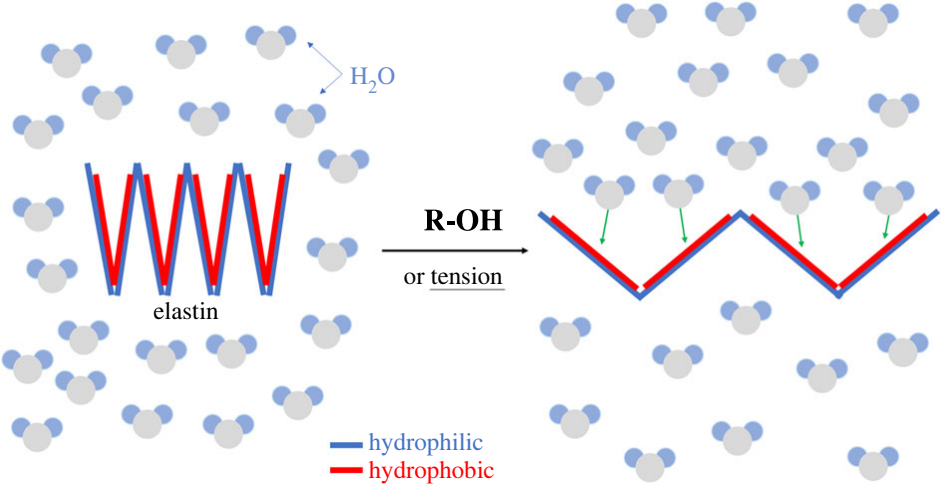
Schematic illustration for the hypothesized cause of elastin cleavage. An illustration for the cause of elastin cleavage seen in this study based on an ‘unfolding’ mechanism. The elastin protein, displayed as a zig-zag chain containing the hydrophilic (blue lines) and hydrophobic (red lines) regions, exists in a ‘folded’ conformation (left) in a hydrophilic environment (i.e. the *in vivo* condition). The addition of alcohols or tension would unfold the chain, thus exposing the hydrophobic regions to the attack of hydrolysis.

To understand the cause of elastin cleavage due to stress relaxation, several theories of the origin of the elastin's elasticity have been proposed [51]. Among them, the hydrophobic effect is the most well-known theory which took the unique hydrophobic feature of elastin into account and explained the reason for the requirement of water for generating elasticity. Because elastin is composed of multiple hydrophilic and hydrophobic domains, under its relaxed state *in vivo*, the polypeptide self-arranges its conformation (folding) to minimize its hydrophobic regions exposed to the hydrophilic environment and maximize its hydrophobic–hydrophobic interactions to achieve the lowest free energy state. As tension is applied, the extended state (unfolding) of the elastin leads to increased hydrophobic–hydrophilic interactions (solvent-accessible hydrophobic regions) and decreased hydrophobic–hydrophobic interactions resulting in a higher free energy state which gives rise to its elasticity. This folding mechanism of elastin not only produces its elasticity, but also contributes to its high resilience to hydrolytic degradation [33–35]. Urry *et al*. studied the effect of conversion of hydrophobic regions into polar residue on hydrophobic folding using elastin-like polypeptides and concluded that the loss of hydrophobic regions causes unfolding and makes the polypeptide more susceptible to proteolysis [34]. Studies on hydrolysis or solubilization of mature elastin using the hot alkaline method [35,52] also discovered the importance of unfolding the hydrophobic regions to increasing the efficiency of hydrolysis. With the addition of an organic solvent (e.g. alcohols) to the reaction solution, the hydrophobic regions of elastin were unfolded and exposed to hydrolytic attack resulting in increased reaction rate and decreased reaction temperature. For the same reason, as shown in figure 11, the stress relaxation treatment would stretch the elastin, exposing the hydrophobic region for several hours for hydrolysis to occur. The time-dependency of elastin cleavage also supports this hypothesis, as a tendon with stress relaxation time = 0 (0 h) produced no elastin cleavage. Studies have shown that elastin can withstand a tensile strain of more than 100% [53]; hence, the cause of cleavage was unlikely due to mechanical fracture but hydrolytic degradation which progresses over time. One possible location at which this hydrolytic degradation took place is the hydrophobic disulfide bonding, which is responsible for the self-assembly nature and the elastin's interaction with the microfibril scaffold of elastic fibres [33]. Existing studies have observed a stress-induced reduction of the disulfide bonds [54,55]. Under tensile forces from 0 to 2 nN on a single disulfide bond, a S_N_2 reaction, in which the hydroxide ion attacks the sulfur atom, is promoted due to a notable decrease of the activation free energy from 27 kcal mol^−1^ to about 11 kcal mol^−1^ without the addition of alkali or heat to the reaction [56,57].

In the *in vivo* experiment, the decreased levels of both crimp angle reduction (−85.1% and −87.6% for GT and SDFT, respectively) (figure 5*b*) and cleaved elastin (−36.6% and −60.4% for GT and SDFT, respectively) (figure 7*b*) from day 1 to day 7 indicate restoration of crimp and remodelling of elastin in the ECM. Since these ECM damages are linked to the tendon lengthening, restoration of these damages is a positive sign of healing. The increase in elastin level in the rabbit tendon ECM (figure 8*a*) and the upregulation of both ELN and COL1A1 expressions from the *in vitro* cell culture (figure 8*b*) also suggest the occurrence of healing.

However, elastin exists not only in the matrix of a tendon fascicle (FM) but also in blood vessels [32]. Since elastin comprises up to 50% of the dry weight of blood vessels [58], the increase in elastin fluorescence level on day 7 might partially come from the newly formed blood vessels. This speculation is verified by the H&E staining performed on the rabbit CCTs. Image analysis discovered an increase of vascularity (Δ*V*) over 80% within the tendon FM soon after 24 h of stress relaxation. However, blood vessels are rarely seen within the FM in a healthy tendon, as tendons are known to be relatively avascular. This neovascularization was likely a result of increased need for nutrient supply. As the tensile strain was applied along the long axis of the tendon, a compressive strain perpendicular to the axis would be produced by the Poisson effect. This compressive strain would decrease the inter-fascicular and the inter-fibre distance, generate internal pressure, cause fluid exudation, and hinder blood flow [59,60]. Therefore, neovascularization was a result of tendon adaptation to the mechanical strain. Another cause of increased vascularity was due to the increased cellular activities to remodel/repair the tendon ECM. Several studies [61,62] have observed increased vascularity at the damage site (e.g. tenotomy, division, suture) of tendons to aid healing and recovery from the damage. In this work, the recovery processes from the ECM micro-damages including crimp angle reduction (figure 4) and elastin cleavage (figure 6) would require remodelling of elastin and collagen. These processes involve cellular migration and proliferation (figure 10*b*), enzymatic degradation of dysfunctional ECM components, and synthesis of new units of these components. All of them consume energy and nutrients [63], resulting in the formation of new blood vessels as a response/adaptation to the reparative processes. The cellular migration/infiltration behaviour, although could provide implications for cell–ECM interactions, was not examined in the *in vitro* study. In addition, a three-dimensional system offered by a tendon explant rather than a two-dimensional decellularized scaffold seeded with tenocytes can offer a more realistic cell–ECM environment and a more complex stress tensor under a uniaxial stretch assuming the homogeneity of nutrient supply can be achieved. Future work should consider repeating this study using an improved three-dimensional system. Lastly, the EDPs generated due to elastin cleavage were also reported to promote angiogenesis [64,65]. While some studies speculate the existence of neovascularization is associated with chronic inflammation or tendinopathy [66,67], it is unlikely to be the case in this study. First, a time length of 7 days is not sufficient to develop chronic tendinopathy [25,68–70]. Second, tissue repair was present during the mechanical treatment as demonstrated from the restoration of crimp angle (figure 5*b*) and ameliorated elastin cleavage level (figure 7*b*). Third, chronic tendinopathy has a low incidence rate in young tendons [71,72].

However, despite the features of healing and recovery mentioned above, inflammation in the tendon ECM (figure 9) persisted even after 7 days, suggesting one week was not enough to fully recover a tendon from the stress relaxation. Furthermore, the increase of vascularity may result in the loss of mechanical strength and function of the collagen-dominant ECM. Compared to the elastic moduli of tendon fascicles, the moduli of blood vessels are much lower [73,74]. Wiesinger *et al*. [75] discovered a reduced elastic modulus in human patellar tendons exhibiting neovascularization. The presence of blood vessels in the FM has been reported to be the source of pain in patellar tendons of jumping athletes [76]. A treatment of the Ponseti method often consists of up to 6 casts to fully correct a clubfoot. How subsequent casts affect the tissue health and recovery are critical questions to answer for future studies. Moreover, in addition to tendons and ligaments, other tissues such as nerves and blood vessels are being strained. Additional studies focused on these tissues responding to stretching are important to improve the treatment. Lastly, although the case of tendinopathy was unlikely in young tendons, treating cases of clubfoot relapse or neglected clubfoot clinically often involves repeated casting in older children [77–79]. Hence, further studies should consider researching the same question on older subjects.

One limitation of the study is the lower power values (electronic supplementary material, table S2) of the rabbit GT results including cleaved elastin ratio (figure 7*b*), elastin fluorescence level (figure 8*a*), and vascularity level (figure 10*a*). Particularly, type II errors could exist in elastin fluorescence level and vascularity level of GT as no statistical difference was found. A future experiment with a rabbit number of 16 per group is required to achieve an 80% power. Another limitation lies in the histological analysis where one section was stained for each tendon of each animal. This limitation is due to the small size of each tendon sample retrieved from each young rabbit, and the majority of a sample was used in IHC and SEM. Future work needs to analyse more histology sections.

## 5. Conclusion

Based on the *ex vivo* results derived from the stress-relaxed DDFTs, time-dependent levels of tendon lengthening and ECM alterations including crimp angle reduction and elastin cleavage were discovered. This time-dependency showing the maximum lengthening of the tendon could be achieved in less than 24 h may support the accelerated protocols proposed by existing work [29,80]. The mechanism behind this lengthening is based on the reduction of crimp angle caused by elastin cleavage. Signs of recovery including the restoration of crimp, alleviated cleaved elastin level, and increased elastin level after 7 days of treatment compared to day 1 were observed from the *in vivo* results. In addition, the increased vascularity in the ECM was likely to be an adaptation from the tendon to supply the recovery.

## Data Availability

All data can be accessed via the following link: https://doi.org/10.5061/dryad.0rxwdbs43. The data are provided in electronic supplementary material [81].
